# A novel mathematical framework for pedigree-based calculation of Y-STR match probabilities

**DOI:** 10.1038/s41598-025-98644-2

**Published:** 2025-04-26

**Authors:** Amke Caliebe, Dion Zandstra, Arwin Ralf, Manfred Kayser, Michael Krawczak

**Affiliations:** 1 Institute of Medical Informatics and Statistics, Kiel University, University Hospital Schleswig-Holstein, Brunswiker Straße 10, 24105 Kiel, Germany; 2 Department of Genetic Identification, Erasmus MC University Medical Center Rotterdam, Rotterdam, The Netherlands; 3 Present Address: Department of Pathology and Clinical Bioinformatics, Erasmus MC University Medical Center Rotterdam, Rotterdam, The Netherlands

**Keywords:** Y-STR, Importance sampling, Suspect population, Simulation, DNA profile, Biological trace, Sexual assault, Forensic genetics, Genetics, Haplotypes

## Abstract

Y-chromosomal short tandem repeat (Y-STR) markers are routinely used in forensic casework to identify male donors of biological traces left at crime scenes, particularly in sexual assault cases. However, the evidential value of a match between the Y-STR profile of a trace and a potential donor, usually a crime suspect, is difficult to quantify, and the common albeit inappropriate practise to equate Y-STR match probabilities with Y-STR profile frequencies estimated from population databases has been subject to scientific debate for decades. As a solution to this long-standing problem, we suggest an alternative approach to the calculation of Y-STR match probabilities that involves splitting the group of potential donors other than the suspect into two: (i) his close male relatives (termed his ‘pedigree’) and (ii) all other males. While an upper limit to the match probability is easily calculated for the second group, it is computationally challenging to derive for the first. We therefore developed a mathematical framework that uses importance sampling to reconstruct and evaluate the Y-STR profiles of untyped members of the suspect’s pedigree by way of simulation. Extensive testing with elementary pedigrees of different structure and complexity confirmed that both, the framework and its Python-based software implementation yield match probability estimates that approximate well the correct analytical results, depending upon the number of simulations performed. Our methodology thus facilitates a more appropriate and valid solution to the long-standing problem of interpreting Y-STR profile matches in forensic casework.

## Introduction

A main goal of forensic genetics is to aid in solving crime cases by identifying the donors of human biological traces left at crime scenes. To this end, DNA profiles comprising short tandem repeat (STR) markers are usually generated from the trace material and subjected to population database searches, or compared directly to the DNA profiles of some individuals of interest, usually including a crime suspect^1,2^. In addition to a multitude of autosomal markers, STRs located in the non-recombining part of the human Y-chromosome have been used for this purpose for more than 30 years^3–5^. Since these Y-STRs are male-specific, they are particularly useful in cases involving male-female DNA mixtures, such as sexual assaults, where often only a Y-STR profile, but no autosomal STR profile, can be obtained of the male contributor^6,7^. However, while autosomal STR profiles usually allow both the inclusion of a suspect as a trace donor (by a complete match at all STRs of the suspect and trace DNA profiles) or their exclusion (by a large number of mismatches), the value of Y-STR profiling in forensic practice is still attributed mainly to a high chance of successful exclusion. There are two main reasons for this.

First, owing to the lack of meiotic recombination and the low to moderate mutation rates of standard Y-STRs used in forensics^8^, a suspect will typically share his Y-STR profile with several of his close male relatives. This implies that, based upon a match between the suspect and trace Y-STR profiles alone, donorship cannot be narrowed down to the suspect with sufficient certainty unless his male relatives are explicitly excluded by non-genetic evidence^6,9,10^. Second, the evidential value of a match between two Y-STR profiles is difficult to quantify because the so-called ‘product rule’ for estimating the population frequency of a profile, which may be justified for autosomal STRs, is not valid for Y-STRs^11^. As long as the pairwise genetic distances between autosomal STRs are large, meiotic recombination rapidly breaks up their haplotypes so that the population frequency of a given profile can be estimated by multiplying the estimated genotype frequencies of its constituent STRs (hence the term ‘product rule’). Y-STRs located in the non-recombining part of the Y chromosome, in contrast, are perfectly genetically linked, which means that their haplotypes are inherited intact along male lineages and can only change by mutation. Therefore, the population frequencies of Y-STR profiles would have to be estimated directly from databases deemed somehow ‘representative’ of the population(s) from which they were sampled^12^. The degree to which such representativeness is achievable, both in terms of size and geographic or ethnic coverage, is however still a matter of scientific debate^7,13^.

The population frequency of a DNA profile is often inappropriately equated to the probability of a complete match (or ‘match probability’, for short) between the DNA profile of a suspect and that of a trace, given that the suspect is not the trace donor. Evidently, ‘population’ in this context must mean the population of plausible alternative suspects and not a certain database population^14^. This distinction may be of minor relevance for autosomal STRs because meiotic recombination and Mendel’s law of independent segregation effectively taper genetic similarity even between close relatives^14^. With Y-STRs, however, the frequency of a profile in a population database of mostly unrelated men (as all currently available Y-STR databases are) is likely to underestimate the match probability in any realistic ‘suspect population’^15^. The reason for this anti-conservativeness is simply that the suspect population will typically include patrilineal male relatives of the suspect who carry the very same Y-STR profile as the suspect, and who are very likely underrepresented in a population database. Consequently, the forensic genetics community has not yet reached a consensus about the statistical interpretation of Y-STR haplotype matches, despite decades of practical use of these genetic markers in routine forensic casework^7^.

For at least 10 years now, the limitations posed to the forensic use of Y-STRs by the high chance of haplotype sharing between male relatives have started to be overcome by the use of so-called ‘rapidly-mutating Y-STRs’ (RM Y-STRs)^16^. RM Y-STRs are characterized by mutation rates that are a magnitude higher, on average, than that of most standard Y-STRs hitherto used in forensic genetics^16–19^. For this reason, RM Y-STRs have much greater potential to pinpoint male trace donors by the exclusion even of close male relatives, based upon mismatching DNA profiles. For example, use of the 30 marker RMplex kit (which includes all 26 currently known RM Y-STRs) allows genetic differentiation between individuals in 43% of father-son pairs, 66% of pairs of brothers, and 76% of uncle-nephew pairs^20^. At the same time, however, the greater diversity of RM Y-STR haplotypes, compared to those of standard Y-STRs, exacerbates the problem of obtaining sensible estimates of match probabilities from population databases in the first place.

One possible solution to the above conundrum would be to hypothetically split the suspect population in a given case into two, namely (i) the immediate patrilineal male relatives of the suspect (henceforth referred to as the suspect’s ‘pedigree’) and (ii) all remaining alternative suspects, who would then be characterized by a certain maximum degree of blood relatedness to the suspect. In the second sub-population, an upper limit to the match probability can be calculated from the marker-specific mutation rates and the minimum number of meioses separating its members from the suspect^14^. In the suspect’s (male) pedigree, by contrast, the match probability can be estimated with great accuracy from the pedigree structure and the Y-STR profiles (if any) of members other than the suspect. Since the match probability in the overall suspect population is limited by the prior-weighted sum of the two figures, the maximum of the latter would then provide a conservative upper limit to the former.

Here, we propose a novel mathematical framework for the estimation of Y-STR haplotype match probabilities in male pedigrees comprising some genotyped members plus all untyped descendants of the most recent common ancestor (MRCA) of the former. The method employs importance sampling to reconstruct and evaluate the haplotypes of the untyped pedigree members, drawing upon the pedigree structure and the mutation rates of the Y-STRs of interest. For the reasons outlined above, our framework will be particularly useful in forensic genetic analyses using RM Y-STRs. What is more, in some countries where Y-STRs are routinely typed in criminal casework, including the Netherlands, the investigative authorities often also collect the necessary information on the pedigree of the suspect if he matches the Y-STR profile of a crime scene trace.

## Results

### Set-up and goal

**Fig. 1:**
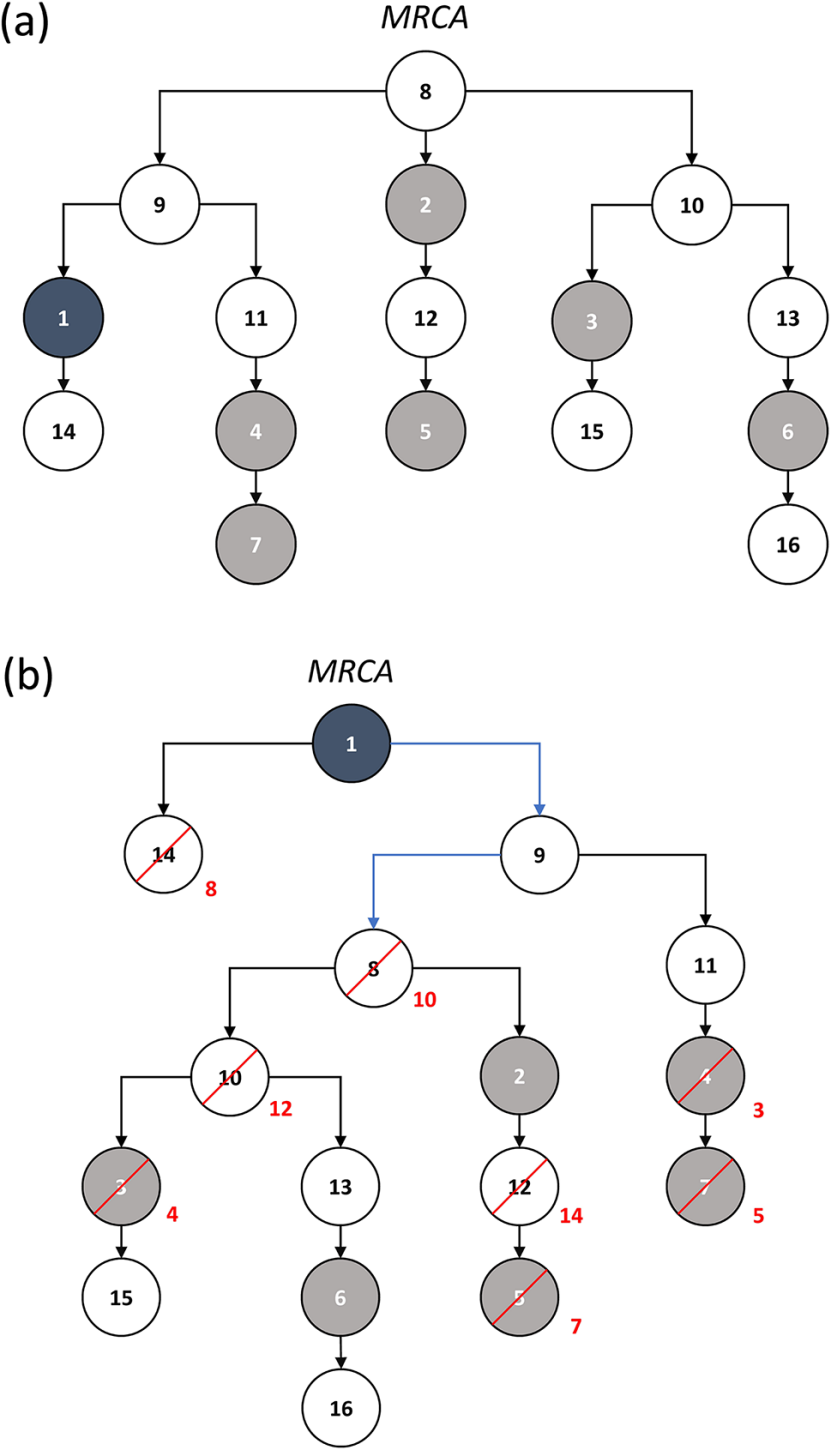
(**a**) Example pedigree comprising 16 patrilineally related males. Males with known haplotypes are indicated by solid symbols, including the suspect who is additionally highlighted in black. MRCA: most recent common ancestor. (**b**) Rearranged and renumbered (red numbers) pedigree, turning the suspect into the MRCA. The two inverted father-son relationships are marked by blue coloring. For details: see text.

For a pedigree comprising n patrilineally related males, we denote the random vector of their Y-STR haplotypes by H=(H_1_,…,H_n_). Let P be the underlying probability measure, i.e. the probability of the n males carrying a particular haplotype combination h=(h_1_,…,h_n_) equals P(H = h). Without loss of generality, we may assume (i) that the first male in a given case is the suspect and (ii) that the first k haplotypes are known while the remaining n-k haplotypes are unknown (Fig. 1a). For convenience, we denote the vector (h_1_,…,h_k_) of known haplotypes by h^v^ and the random vector (H_k+1_,…,H_n_) of unknown haplotypes by H^u^.

If m(H^u^) is the (random) number of unknown haplotypes that match the suspect haplotype h_1_, the goal of forensic genetic analysis is to derive the conditional probability p_x_=P(m(H^u^) = x|h^v^) for any integer x with 0 < x ≤ n-k.

### Importance sampling

The exact value of p_x_ is difficult to determine analytically and, therefore, is better approximated by repeated simulation of H^u^, conditional upon h^v^. From the simulated vectors h^u^, probability p_x_ can be estimated as the relative number of times m(h^u^) = x was observed. However, a classical Monte Carlo approach, i.e. simulation of H^u^ according to P, is difficult to implement because the algorithm would have to account for the variable alternation of known and unknown haplotypes along individual albeit interdependent patrilines. Moreover, a Monte Carlo approach is also likely to be highly inefficient because m(h^u^) would equal zero most of the time if p_x_ < < 1, as will often be the case in forensic practice. A large number of simulations would then be required to estimate p_x_ with sufficient accuracy.

Alternatively, H^u^ can be simulated by way of importance sampling^21^ using a different probability measure Q, called the ‘proposal distribution’, which is characterized by q_x_=Q(m(H^u^) = x|h^v^) > > p_x_. Rather than estimating p_x_ by the relative number of times m(h^u^) = x occurred, one then has to sum up the probability ratios, or ‘proposal weights’, P(H^u^=h^u^|h^v^)/Q(H^u^=h^u^|h^v^) over simulations that fulfil m(h^u^) = x, and divide the final sum by the total number of simulations.

### Change of pedigree representation

A patrilineal pedigree is a directed graph comprising nodes that correspond to individuals and edges that correspond to father-son relationships. At the root of the graph is the most recent common ancestor (MRCA) of the pedigree members so that the tree can be viewed as a set of rope-connected balls suspended from the MRCA (Fig. 1a). The structure of the pedigree is fully described by the pairs of identifiers signifying a pedigree member and his father, or by the information that the member in question is the MRCA.

Generalized calculation of the proposal weights is hampered by the case-specificity, and hence variability, of the suspect’s position in the pedigree. However, the mutation process of Y-STRs can be assumed to be more or less symmetrical, i.e. the rate µ(A, B) at which haplotype A mutates to haplotype B is roughly the same as the rate µ(B, A) at which B mutates to A^19,22,23^. Without loss of generality, the pedigree can therefore be rearranged such that the suspect becomes the MRCA (Fig. 1b). To this end, all father-son relationships along the lineage between the suspect and the MRCA have to be inverted. Figuratively speaking, the set of rope-connected balls is suspended from the suspect rather than the MRCA. Also, without loss of generality, the identifiers of the pedigree members can be modified so that, within the group of either known or unknown haplotypes, numbering of individuals increases by generation, starting from the suspect as the new MRCA (Fig. 1b).

### The proposal distribution

For the importance sampling-based simulation of H^u^, let 0 < x ≤ n-k be a fixed target number of matches between unknown haplotypes and the suspect haplotype h_1_. Each simulation proceeds in two parts: First, randomly draw x unknown haplotypes and set them equal to h_1_. In the second part, iteratively draw the individual with the smallest identifier, say i, from the residual set of unknown haplotypes. Let π(i) be the identifier of his father. Then simulate h_i_ from h_π(i)_ according to the underlying mutation rates µ(h_π(i)_,⋅) and remove H_i_ from the residual set of unknown haplotypes. The second part of the algorithm is repeated until all unknown haplotypes have been simulated and the residual set of unknown haplotypes is empty.

In parallel to the simulation of h^u^, the denominator Q(H^u^=h^u^|h^v^) of the (simulation-specific) proposal weight is calculated as follows: Before the simulation, set the denominator equal to the inverse of the binomial coefficient bin(x, n-k). Then multiply the denominator with the corresponding mutation rate µ(h_π(i)_,h_i_) in each step of the simulation which, at the end of the simulation, results in the correct denominator.

For the numerator P(H^u^=h^u^|h^v^) of the proposal weight, calculate the product of the µ(h_π(i)_,h_i_) values of all father-son pairs once the simulation is finished (i.e. when all unknown haplotypes have been simulated and are thus known). To obtain numerator P(H^u^=h^u^|h^v^), the product still has to be divided by P(h^v^), which is estimated by simulation as well (see below). However, since P(h^v^) depends only upon known haplotypes, it needs to be calculated only once per pedigree.

### Estimation of P(h^v^)

The simulation process to estimate P(h^v^) is very similar to the one described above, but with two main modifications: First, no unknown haplotypes are a priori set equal to the suspect haplotype (i.e. x = 0). Second, at the end of each simulation, the µ(h_π(i)_,h_i_) values are multiplied only over those father-son pairs (π(i), i) for which i ≤ k, i.e. for which the haplotype of the son was known and was thus not simulated. The average of these products, taken over all simulations, provides a valid estimate of P(h^v^).

The last assertion can be justified as follows: In principle, P(h^v^) could be estimated by the relative number of times H^v^ equals h^v^ in a joint Monte Carlo simulation of H^v^ and H^u^. However, such matches would be extremely rare, rendering the process highly inefficient. Therefore, in this case, too, it would be sensible to estimate P(h^v^) by importance sampling. Maximum efficiency of the process is achieved if, in every simulation, H^v^ is set equal to h^v^ and only H^u^ is simulated, conditional on h^v^. The sought-after P(h^v^) is then estimated by the average of the proposal weights, taken over all simulations. For a given simulation, the numerator of the proposal weight equals the product of the µ(h_π(i)_,h_i_) values for all father-son pairs. The denominator, in contrast, comprises only µ(h_π(i)_,h_i_) values for pairs involved in the simulation of h^u^. Reducing the ratio of the two expression thus leaves the product of the µ(h_π(i)_,h_i_) values for pairs not involved in the simulation (i.e. for which i ≤ k) as the sought-after proposal weight.

### Software test

**Fig. 2:**
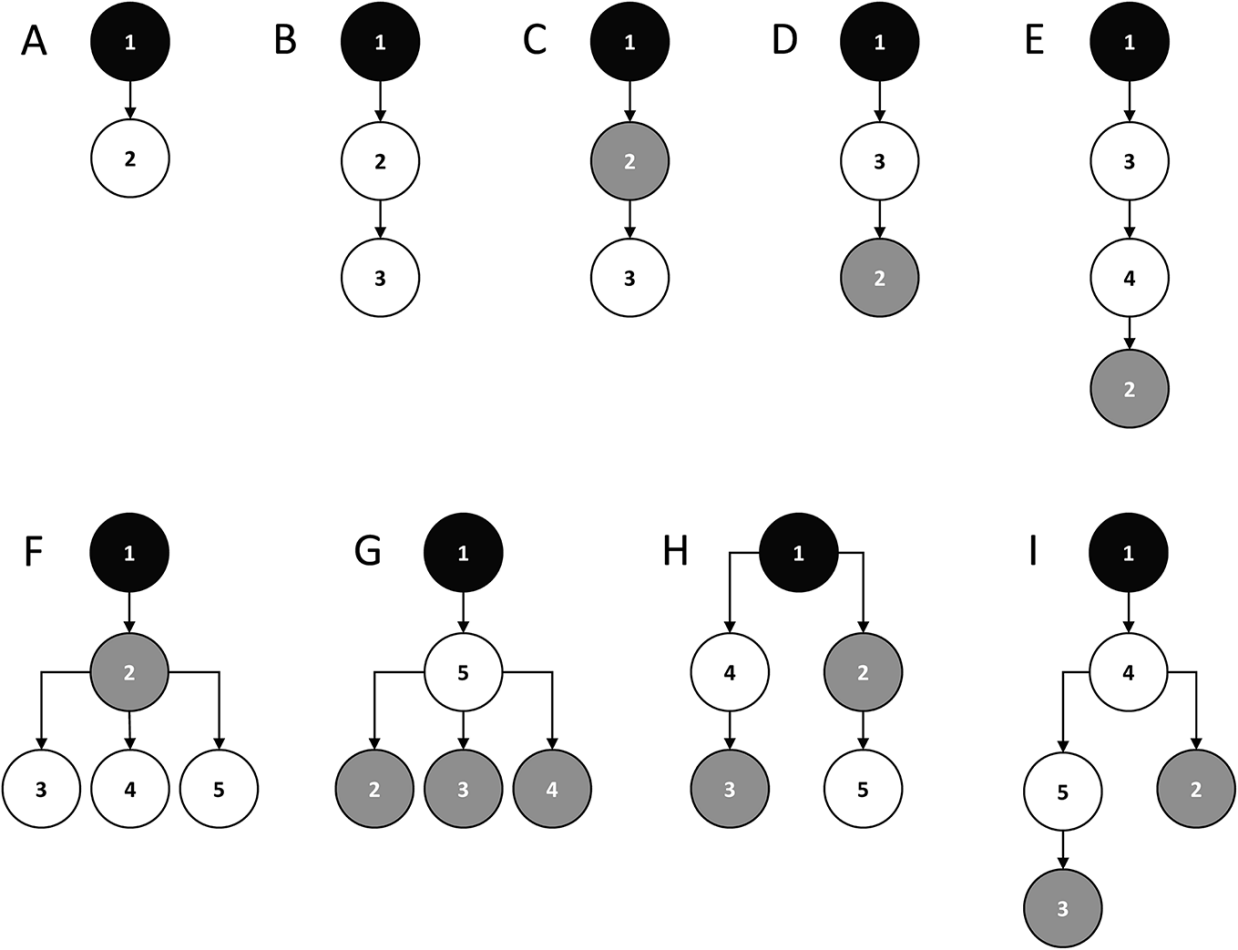
Generic elementary pedigrees comprising typed (solid symbols) and untyped males (open symbols), as used for testing the importance sampling framework and its Python implementation. In all pedigrees, the suspect (black symbol) is the most recent common ancestor of all male family members.

The importance sampling framework described above was implemented in a customized Python script (version 3.9), and both the framework and the software were tested for a set of generic elementary pedigrees (Fig. 2) and a single Y-STR with integer-valued alleles (see Methods for further details). In the tests, one of 34 possible allele assignments to typed pedigree members (Table 1) and one of three different mutation rates µ (0.1, 0.02, or 0.005 per meiosis) were considered.

**Table 1:** Computation of match probabilities in elementary pedigrees.

Pedigree^a^	Allele Assignment^a^				P(h^v^)	p_x_ × P(h^v^)^b^		
	ID	2	3	4		x = 1	x = 2	x = 3
A	A01	n.a.	n.a.	n.a.	1	1-2z	n.a.	n.a.
B	B01				1	2z(1-z)	(1-2z)^2^	
C	C01	0			1-2z	(1-2z)^2^	n.a.	
	C02	+ 1			z	z^2^		
D	D01	0			2z^2^+(1-2z)^2^	(1-2z)^2^		
	D02	+ 1			2z(1-2z)	z(1-2z)		
E	E01	0			6z^2^(1-2z)+(1-2z)^3^	4z^2^(1-2z)	(1-2z)^3^	
	E02	+ 1			z^3^ + 3z(1-2z)^2^	z^3^ + z(1-2z)^2^	z(1-2z)^2^	
	E03	+ 2			3z^2^(1-2z)	z^2^(1-2z)	0	
F	F01	0			1-2z	12z^2^(1-2z)^2^	6z(1-2z)^3^	(1-2z)^4^
	F02	+ 1			z	3z^2^(1-z)^2^	3z^3^(1-z)	z^4^
G	G01	0	0	0	z^4^+(1-2z)^4^	(1-2z)^4^	n.a	n.a.
	G02	+ 1	0	0	z^3^(1-2z) + z(1-2z)^3^	z(1-2z)^3^		
	G03	+ 1	+ 1	0	2z^2^(1-2z)^2^	z^2^(1-2z)^2^		
	G04	+ 1	+ 1	+ 1	z^3^(1-2z) + z(1-2z)^3^	z^3^(1-2z)		
H	H01	0	0	n.a.	4z^3^(1-2z) + 2z(1-z)(1-2z)^2^+(1-2z)^4^	2z(1-z)(1-2z)^2^	(1-2z)^4^	n.a.
	H02	0	+ 1		2z(1-2z)^2^	z(1-2z)^2^	z(1-2z)^3^	
	H03	0	+ 2		z^2^(1-2z)	z^2^(1-2z)^2^	0	
	H04	+ 1	0		2z^3^ + z(1-2z)^2^	z(1-z)(1-2z)^2^+2z^4^	z^2^(1-2z)^2^	
	H05	+ 1	+ 1		2z^2^(1-2z)	z^2^(1-2z)	z^3^(1-2z)	
	H06	+ 1	+ 2		z^3^	z^4^	0	
I	I01	0	0	n.a.	4z^3^(1-2z) + 2z^2^(1-2z)^2^+(1-2z)^4^	2z^3^(1-2z) + 2z^2^(1-2z)^2^	(1-2z)^4^	n.a.
	I02	0	+ 1		2z^4^ + z^2^(1-2z)^2^+2z(1-2z)^3^	2z^4^ + z(1-2z)^3^	z(1-2z)^3^	
	I03	0	+ 2		2z^3^(1-2z) + z^2^(1-2z)^2^	z^2^(1-2z)^2^	0	
	I04	0	+ 3		z^4^	0	0	
	I05	+ 1	0		z^3^(1-2z) + 2z^2^(1-2z)^2^+z(1-2z)^3^	z^3^(1-2z) + z^2^(1-2z)^2^	z(1-2z)^3^	
	I06	+ 1	+ 1		z^3^(1-2z) + 2z^2^(1-2z)^2^+z(1-2z)^3^	z^3^(1-2z) + z^2^(1-2z)^2^	z^2^(1-2z)^2^	
	I07	+ 1	+ 2		z^3^(1-2z) + 2z^2^(1-2z)^2^	z^3^(1-2z)	0	
	I08	+ 1	+ 3		z^3^(1-2z)	0	0	
	I09	+ 2	0		2z^3^(1-2z)	z^3^(1-2z)	0	
	I10	+ 2	+ 1		2z^4^ + z^2^(1-2z)^2^	z^4^	0	
	I11	+ 2	+ 2		2z^3^(1-2z)	0	0	
	I12	+ 2	+ 3		z^4^	0	0	

^a^Pedigrees and males with known alleles are labelled according to Fig. 2. ^b^For a given value of x (x = 1, 2, 3), match probability p_x_ is obtained by dividing the entry in the respective column by the entry in column ‘P(h^v^)’. P(h^v^): probability of allele assignment, given that the suspect has allele 0; p_x_: probability of x allelic matches between the suspect and untyped pedigree members; z: unidirectional mutation rate µ/2; n.a.: not applicable.

For 89 of the ensuing 3 × 34 = 102 test scenarios, including all scenarios with µ = 0.1, both the average and the standard deviation of the percentage difference between exact and simulated P(h^v^) value were smaller than 10% when 100,000 simulations were carried out per test (Table 2a). For another five scenarios, the same level of accuracy was achieved after increasing the number of simulations per test to 1 million. The eight remaining scenarios all involved pedigree I and either µ = 0.02 (two scenarios) or µ = 0.005 (six scenarios). The number of simulations performed per second on the IT platform used (see Methods) varied between ~ 4500 (assignment I11) and ~ 9500 (assignment A01), with a clear gradient following the complexity of both the pedigree and the allele assignment. For p_1_, a total of 10 scenarios (three with µ = 0.02, seven with µ = 0.005) called for an increase of the simulation number to 1 million, which still left three scenarios (all with µ = 0.005) with a standard deviation > 10%, but none with an average > 10%, after the increase (Table 2b). For p_2_ and p_3_, all relevant scenarios yielded an average and a standard deviation ≤ 10% at 100,000 simulations per test (Table 2c and d).

**Table 2:** Accuracy of importance sampling-based probability estimation.

Allele assignment ID	Mutation rate (µ)								
	**0.1**			0.02			0.005		
	Exact	Perc. diff^a^	Sim	Exact	Perc. diff^a^	Sim	Exact	Perc. diff^a^	Sim
(a) Probability P(h^v^) of allele assignment									
A01	1	< 0.05 (< 0.05)	9622	1	< 0.05 (< 0.05)	9555	1	< 0.05 (< 0.05)	9474
B01	1	< 0.05 (< 0.05)	7084	1	< 0.05 (< 0.05)	7093	1	< 0.05 (< 0.05)	7052
C01	0.90	< 0.05 (< 0.05)	6968	0.98	< 0.05 (< 0.05)	6956	0.995	< 0.05 (< 0.05)	6987
C02	0.050	< 0.05 (< 0.05)	6983	0.010	< 0.05 (< 0.05)	6943	0.0025	< 0.05 (< 0.05)	6928
D01	0.82	0.1 (0.1)	6979	0.96	< 0.05 (< 0.05)	6998	0.99	< 0.05 (< 0.05)	6952
D02	0.090	0.2 (0.6)	6886	0.020	-0.4 (1.3)	6945	0.0050	-0.7 (1.8)	6917
E01	0.74	< 0.05 (0.1)	5585	0.94	< 0.05 (0.1)	5602	0.99	< 0.05 (< 0.05)	5532
E02	0.12	0.2 (0.8)	5551	0.029	0.1 (0.6)	5590	0.0074	-1.2 (1.9)	5556
E03	0.0068	0.8 (2.2)	5614	2.9 × 10^–4^	-5.6 (9.8)	5570	1.9 × 10^–5^	*4.5 (5.3)*	5587
F01	0.90	< 0.05 (< 0.05)	4734	0.98	< 0.05 (< 0.05)	4681	0.995	< 0.05 (< 0.05)	4684
F02	0.050	< 0.05 (< 0.05)	4765	0.010	< 0.05 (< 0.05)	4722	0.0025	< 0.05 (< 0.05)	4680
G01	0.66	< 0.05 (0.1)	4692	0.92	< 0.05 (< 0.05)	4681	0.98	< 0.05 (< 0.05)	4612
G02	0.037	0.1 (0.1)	4683	0.0094	< 0.05 (< 0.05)	4708	0.0025	< 0.05 (< 0.05)	4627
G03	0.0041	-0.4 (0.5)	4707	1.9 × 10^–4^	0.2 (1.9)	4607	1.2 × 10^–5^	-1.0 (3.9)	4629
G04	0.037	0.3 (1.4)	4709	0.0094	-0.4 (3.0)	4623	0.0025	-5.4 (4.6)	4627
H01	0.73	< 0.05 (0.1)	4697	0.94	< 0.05 (< 0.05)	4635	0.99	< 0.05 (< 0.05)	4571
H02	0.081	0.2 (0.9)	4678	0.019	-0.9 (1.3)	4591	0.0050	-1.4 (2.9)	4584
H03	0.0023	-0.2 (1.0)	4669	9.8 × 10^–5^	-1.6 (3.1)	4608	6.2 × 10^–6^	-2.0 (7.0)	4594
H04	0.041	< 0.05 (0.1)	4706	0.0096	< 0.05 (< 0.05)	4575	0.0025	< 0.05 (< 0.05)	4559
H05	0.0045	-0.2 (0.5)	4677	2.0 × 10^–4^	-0.6 (2.2)	4610	1.2 × 10^–5^	1.7 (2.8)	4577
H06	1.3 × 10^–4^	-0.6 (1.3)	4701	1.0 × 10^–6^	-1.2 (3.3)	4640	1.6 × 10^–8^	0.6 (6.4)	4588
I01	0.66	< 0.05 (0.1)	4675	0.92	< 0.05 (0.1)	4634	0.98	< 0.05 (< 0.05)	4623
I02	0.075	0.1 (0.6)	4669	0.019	-0.2 (0.8)	4611	0.0049	-0.4 (3.8)	4602
I03	0.0023	-0.3 (1.1)	4684	9.8 × 10^–5^	< 0.05 (3.8)	4611	6.2 × 10^–6^	< 0.05 (9.5)	4516
I04	6.3 × 10^–6^	-1.1 (7.2)	4689	1.0 × 10^–8^	*-0.1 (10.8)*	4608	3.9 × 10^–11^	*8.8 (47.0)*	4615
I05	0.041	0.2 (0.5)	4668	0.0096	-0.1 (0.3)	4621	0.0025	-0.2 (0.2)	4586
I06	0.041	0.1 (1.0)	4653	0.0096	1.6 (4.1)	4649	0.0025	-3.1 (5.5)	4565
I07	0.0042	0.3 (4.5)	4589	1.9 × 10^–4^	*-0.9 (4.4)*	4614	1.2 × 10^–5^	*-3.4 (19.7)*	4556
I08	1.1 × 10^–4^	-2.4 (7.9)	4611	9.8 × 10^–7^	*5.5 (11.4)*	4634	1.6 × 10^–8^	*10.4 (38.8)*	4500
I09	2.3 × 10^–4^	-1.3 (3.4)	4589	2.0 × 10^–6^	*0.5 (4.7)*	4600	3.1 × 10^–8^	*-4.7 (19.2)*	4524
I10	0.0021	-0.3 (1.6)	4583	9.6 × 10^–5^	-0.3 (2.6)	4564	6.2 × 10^–6^	3.2 (8.8)	4520
I11	2.3 × 10^–4^	0.2 (5.1)	4604	2.0 × 10^–6^	*0.3 (4.9)*	4573	3.1 × 10^–8^	*0.6 (28.9)*	4483
I12	6.3 × 10^–6^	1.4 (5.2)	4600	1.0 × 10^–8^	*-4.4 (7.7)*	4602	3.9 × 10^–11^	*10.4 (44.3)*	4510

Allele assignment ID	Mutation rate (µ)								
	0.1		0.02		0.005				
	Exact	Perc. diff	Exact	Perc. diff	Exact	Perc. diff			
(b) Match probability p_1_									
A01	0.90	< 0.05 (< 0.05)	0.98	< 0.05 (< 0.05)	> 0.99	< 0.05 (< 0.05)			
B01	0.095	0.9 (1.9)	0.020	1.2 (3.3)	0.0050	3.8 (5.7)			
C01	0.90	< 0.05 (< 0.05)	0.98	< 0.05 (< 0.05)	> 0.99	< 0.05 (< 0.05)			
C02	0.050	< 0.05 (< 0.05)	0.010	< 0.05 (< 0.05)	0.0025	< 0.05 (< 0.05)			
D01	> 0.99	-0.1 (0.1)	> 0.99	< 0.05 (< 0.05)	> 0.99	< 0.05 (< 0.05)			
D02	0.50	-0.2 (0.6)	0.50	0.4 (1.3)	0.50	0.7 (1.8)			
E01	0.012	0.4 (1.3)	4.2 × 10^–4^	1.6 (2.2)	2.5 × 10^–5^	-2.4 (2.7)			
E02	0.33	-0.3 (1.5)	0.33	-0.1 (4.8)	0.33	6.0 (6.3)			
E03	0.33	-2.0 (2.3)	0.33	5.9 (8.1)	0.33	*-5.0 (5.9)*			
F01	0.027	-1.2 (2.2)	0.0012	*1.6 (4.1)*	7.5 × 10^–5^	*-0.8 (12.3)*			
F02	0.14	< 0.05 (0.1)	0.029	< 0.05 (< 0.05)	0.0075	< 0.05 (< 0.05)			
G01	> 0.99	< 0.05 (0.1)	> 0.99	< 0.05 (< 0.05)	> 0.99	< 0.05 (< 0.05)			
G02	> 0.99	-0.1 (0.1)	> 0.99	< 0.05 (< 0.05)	> 0.99	< 0.05 (< 0.05)			
G03	0.50	0.4 (0.5)	0.50	-0.1 (1.9)	0.50	1.1 (3.9)			
G04	0.0031	-0.3 (1.4)	1.0 × 10^–4^	0.5 (3.1)	6.3 × 10^–6^	5.9 (4.9)			
H01	0.10	-0.3 (1.2)	0.020	-1.4 (3.2)	0.0050	3.5 (3.3)			
H02	0.50	-0.1 (1.5)	0.50	0.4 (6.1)	0.50	-3.6 (8.5)			
H03	0.90	0.5 (2.3)	0.98	-0.6 (4.9)	> 0.99	*1.6 (3.6)*			
H04	0.94	0.2 (0.4)	0.99	0.1 (0.4)	> 0.99	< 0.05 (0.2)			
H05	0.50	0.1 (0.6)	0.50	0.6 (2.3)	0.50	-1.6 (2.8)			
H06	0.050	-0.2 (1.6)	0.010	0.5 (5.2)	0.0025	*0.4 (2.5)*			
I01	0.0065	-0.3 (1.6)	2.1 × 10^–4^	-2.0 (3.3)	1.3 × 10^–5^	-0.1 (6.6)			
I02	0.49	-0.1 (1.6)	0.50	1.2 (5.1)	0.50	1.8 (8.9)			
I03	0.90	0.3 (2.1)	0.98	1.6 (8.3)	> 0.99	5.0 (9.7)			
I04	0	0	0	0	0	0			
I05	0.053	5.5 (2.3)	0.010	-0.1 (4.2)	0.0025	-2.3 (9.2)			
I06	0.053	-0.5 (2.3)	0.010	-2.1 (5.4)	0.0025	*1.6 (3.0)*			
I07	0.027	0.1 (4.1)	0.0051	*0.6 (4.3)*	0.0013	*7.2 (20.8)*			
I08	0	0	0	0	0	0			
I09	0.50	0.8 (5.0)	0.50	*-0.5 (4.1)*	0.50	*8.3 (19.7)*			
I10	0.0031	0.7 (2.5)	1.0 × 10^–4^	-1.9 (5.7)	6.3 × 10^–6^	-5.0 (8.9)			
I11	0	0	0	0	0	0			
I12	0	0	0	0	0	0			
(c) Match probability p_2_									
B01	0.81	< 0.05 (< 0.05)	0.96	< 0.05 (< 0.05)	0.99	< 0.05 (< 0.05)			
E01	0.98	< 0.05 (0.1)	> 0.99	< 0.05 (0.1)	> 0.99	< 0.05 (< 0.05)			
E02	0.33	-0.2 (0.8)	0.33	-0.1 (0.6)	0.33	1.3 (2.0)			
E03	0	0	0	0	0	0			
F01	0.24	-0.3 (0.9)	0.058	-0.2 (3.3)	0.015	-0.4 (2.5)			
F02	0.0071	< 0.05 (0.1)	3.0 × 10^–4^	< 0.05 (< 0.05)	1.9 × 10^–5^	< 0.05 (< 0.05)			
H01	0.89	< 0.05 (0.1)	0.98	< 0.05 (< 0.05)	0.99	< 0.05 (< 0.05)			
H02	0.45	-0.1 (0.9)	0.49	0.9 (1.4)	0.50	1.5 (3.0)			
H03	0	0	0	0	0	0			
H04	0.050	< 0.05 (0.1)	0.010	< 0.05 (< 0.05)	0.0025	< 0.05 (< 0.05)			
H05	0.025	0.2 (0.5)	0.0050	0.6 (2.2)	0.0013	-1.6 (2.7)			
H06	0	0	0	0	0	0			
I01	0.99	< 0.05 (0.1)	> 0.99	< 0.05 (0.1)	> 0.99	< 0.05 (< 0.05)			
I02	0.49	-0.1 (0.6)	0.50	0.2 (0.8)	0.50	0.6 (3.7)			
I03	0	0	0	0	0	0			
I04	0	0	0	0	0	0			
I05	0.90	-0.2 (0.5)	0.98	0.1 (0.3)	0.99	0.2 (0.2)			
I06	0.050	-0.1 (1.0)	0.010	-1.4 (3.9)	0.0025	3.5 (5.8)			
I07	0	0	0	0	0	0			
I08	0	0	0	0	0	0			
I09	0	0	0	0	0	0			
I10	0	0	0	0	0	0			
I11	0	0	0	0	0	0			
I12	0	0	0	0	0	0			
(d) Match probability p_3_									
F01	0.73	< 0.05 (< 0.05)	0.94	< 0.05 (< 0.05)	0.99	< 0.05 (< 0.05)			
F02	1.3 × 10^–4^	< 0.05 (< 0.05)	1.0 × 10^–6^	< 0.05 (< 0.05)	1.6 × 10^–8^	< 0.05 (< 0.05)			

^a^“< 0.05”: absolute value in question is below 0.05. Exact: exact value according to Table 1. Perc. diff: average percentage difference between exact value and importance sampling-based estimate, taken over 10 tests per combination of mutation rate and allele assignment (standard deviation in brackets), Sim: Average number of simulations performed per second by a Python implementation on a 22-core Intel^®^ Xeon^®^ Gold 6152 CPU. Estimates were based upon 100,000 simulations (normal print) or 1 million simulations (italics) per test.

## Discussion

In the early 1920s, British statistician and geneticist Sir Ronald A. Fisher coined what is today known as the ‘Law of Likelihood’, stating that the likelihood of a hypothesis Z in the light of some observation X is proportional to the probability of observing X when Z is true^24^. This principle is also reflected in the way the results of forensic genetic analyses are incorporated into the process of court decision-making, where the evidential value of the DNA evidence at hand is usually quantified by the likelihood ratio of the two hypotheses, Z_d_ (numerator) and Z_p_ (denominator), respectively, which the defence and the prosecution propose about the foundation of the evidence. More specifically, Z_p_ stipulates that the suspect is the trace donor whereas Z_d_ means that he is not. In the simplest case, when X represents a match between the DNA profiles of a suspect and a biological trace left at a crime scene, the likelihood of Z_p_ equals unity. This implies that the likelihood ratio equals the match probability between trace and suspect DNA profile given that defence hypothesis Z_d_ is true. This agreement explains why the match probability has become a key concern of forensic geneticists aiming to ensure a fair and consistent interpretation of their laboratory results.

Unfortunately, in the case of Y-STRs, these well-meant intentions are compromised by the inadequacy of equating the match probability with the population frequency of the corresponding Y-STR haplotype, particularly if this frequency was estimated from a database of mostly unrelated males. There are two main reasons for this standpoint. For one, it is unlikely that a plausible group of potential donors in a given case does not bear any ethnic, social or geographical relationship to the suspect, which implies that the assumption of non-relatedness inherent to named population databases is anti-conservative in the sense that, on average, it disadvantages the suspect^14^. Second, the utility of a set of Y-STRs in forensic practice depends upon the diversity of its haplotype spectrum. The greater this diversity, the greater is the discriminatory power of the marker set, which has not least driven an enormous expansion of commercial Y-STR kits in the past^6^. However, beyond our general concerns about the use of population databases to infer match probabilities, ever greater diversity of haplotypes leads to ever rarer haplotypes for which the population frequencies are ever more difficult to estimate accurately from databases of limited size.

As far as we know, all previous approaches to calculating match probabilities for Y-STRs were indeed based upon haplotype frequencies derived from population databases of unrelated males. The so-called ‘Discrete Laplace method’^25^ is no exception in this regard. Its popularity is rather due to the fact that it takes population genetic theory explicitly into account and is therefore expected to perform better than other estimation methods, including simple counting in databases and more advanced model-based statistical approaches^26–29^. However, the Discrete Laplace method works well only for Y-STRs with low mutation rates and is known to be anti-conservative for high mutation rates^30,31^. This is particularly unfortunate because, as was mentioned in the Introduction, one way to overcome the problem of reduced informativeness due to extensive Y-chromosomal haplotype sharing between close male relatives is the use of RM Y-STRs for forensic testing^7,18,32^.

The mathematical framework for Y-STR match probability calculation presented here started from the idea of dissecting the defence hypothesis Z_d_ into two disjoint hypotheses, Z_f_ and Z_u_, of which Z_f_ stipulates that the trace donor is an untyped member of the suspect’s (male) pedigree whereas Z_u_ implies that the trace donor does not belong to the pedigree at all. If all typed pedigree members other than the suspect have been excluded from trace donorship by the police, then Z_d_=Z_f_∪Z_u_ and the sought-after match probability under Z_d_ is limited by the maximum product of the match probability under either Z_f_ or Z_u_ and the conditional prior probability of the respective hypothesis, given that Z_d_ is true.

With a large number of RM Y-STRs such as, for example, the 30 RM Y-STRs of the RMplex kit^33^, perfect haplotype matches become rather unlikely between distantly related males as represented by Z_u_. What is more, the corresponding match probabilities do not necessarily have to be estimated, but an exact upper limit could be calculated by accounting for the maximum degree of partilineal relatedness considered for Z_u_ in a given forensic case. This maximum degree of relatedness should be chosen as small as possible but, in practise, would be limited by the level of coverage justifiable during the police investigations. Nevertheless, even with realistic assumptions about the depths of patrilines ascertainable in forensic practice, RM Y-STRs can reduce the maximum match probability under hypothesis Z_u_ to a level that narrows down the task of considering potential alternative trace donors to the known patrilineal male relatives of the suspect. While all members of the suspect’s pedigree should ideally be genotyped to this end, it would be most important to include the father and sons (if any) of the suspect. This is because mismatches between their haplotypes and the haplotype of the suspect would have to be reversed by additional mutations to result in complete matches with more distant relatives, which means that the latter are rather unlikely. In forensic practice, however, only some pedigree members would be available for Y-STR genotyping in a given case while others would not and, at the same time, could not be excluded as trace donors based upon non-genetic evidence. In this situation, deriving the likelihood of hypothesis Z_f_ becomes tantamount to calculating the match probability between the Y-STR profiles of the trace and at least one non-typed member of the suspect’s pedigree.

We developed a novel mathematical framework for the pedigree-based calculation of match probabilities and created and tested a computer implementation of this framework for practical use. We wish to emphasize at this point that our work was explicitly not aimed at achieving the lowest possible match probability in every case because this could easily systematically favour the prosecution over the defence. Instead, we wanted to provide a way out of the incorrect approach to quantifying the evidential value of Y-STR profile matches that equates the latter with frequency estimates from population databases. Our approach yields a valid match probability for the untyped members of a suspect’s pedigree and, if this match probability is large, the evidential value of the Y-STR haplotype match is simply not strong (enough). The police would then have to focus their investigations on reducing the conditional prior probability of defence hypothesis Z_f_, mainly by exonerating additional untyped members of the suspect’s pedigree by typing them or by gathering suitable non-genetic evidence such as place of residence, age, circumstances of living (i.e. travel history), or lack of other investigative information. We are fully aware that our approach does not align with the latest recommendations on the interpretation of Y-STR data in forensic analysis by the International Society of Forensic Genetics (ISFG)^7^. However, relying solely upon low population frequency estimates derived from databases, and at the same time disregarding the family background of suspects, in our view no longer meets agreed standards of fairness and justice.

So far, we have only tested the basic functionality of the computer implementation of our mathematical framework. For this reason, only small generic family trees, which nevertheless are realistic in some real-life cases and also represent the building blocks of more complex pedigrees, and only a single Y-STR were considered. Moreover, the tests covered only three different mutation rates, one of which (µ = 0.1) was unrealistically high for standard Y-STRs, but not necessarily certain RM Y-STRs^19,34^, and which was chosen mainly because it facilitated effective implementation testing. Future work will therefore address the performance of the software under more realistic conditions, including multiple Y-STRs, empirical mutation rates and larger pedigrees. In any case, the fact that all tests of scenarios with µ = 0.1 yielded percentage differences between exact and simulated match probabilities below 10% strongly suggests that the importance sampling framework is valid and that its Python implementation works correctly. Moreover, since a 10-fold increase of the number of simulations per test often resulted in the same level of accuracy for smaller mutations rates as well, it seems advisable in forensic practise to repeat one and the same calculation multiple times and to iteratively adapt the underlying simulation number to the observed outcome variance. We will implement this functionality in a future publicly available version of our software.

One limitation of our approach is that it requires detailed knowledge of the immediate male family background of the suspect. Such information may not always be easy to obtain, particularly in countries where family record keeping is not widespread. Moreover, for the approach to be effective, at least some male relatives would have to be genotyped for the Y-STRs of interest, with their exact number and choice depending upon the actual pedigree structure. Achieving this goal is critically dependent upon the willingness of the relatives to cooperate with the police because, in most legal systems, the enforced genotyping of individuals who are not themselves suspects in a criminal case is difficult, if not impossible.

In the future, we will evaluate the accuracy and performance of the computer implementation of our novel mathematical framework in more detail, including its run-time behaviour on different platforms and under more realistic scenarios regarding pedigree size, marker number and mutation rate. Another focus of our research will be the possibility of multi-copy markers, fractional alleles and the choice of appropriate simulation parameters depending upon the specifics of individual cases. The ultimate goal of our endeavours will be the provision, to the forensic genetic community, of a software tool fit for practical use. However, whether pedigree-based match probabilities, as proposed here, will make their way into routine forensic casework will critically depend upon the openness of the police towards the approach. According to our perception, a certain level of acceptance by the law enforcement agencies is already evident, for example, in the Netherlands, but it is also clear that the necessary changes of practice would entail additional tasks and work for the police. First and foremost, the police would have to gain access to the relevant pedigree information, for example, through municipal archives or church records, and they would have to get permission to use this information for forensic purposes. Here, too, the necessary legal requirements appear to be at least partially met in the Netherlands. Second, the police must be legally entitled and practically able to collect biomaterial from pedigree members other than the suspect for Y-STR testing (see above). Finally, the use of pedigree-based match probabilities would demand some adjustment also on the side of the forensic geneticists. The interpretation of Y-STR genotyping results would no longer rely solely on their expertise and the consultation of population databases but would instead require close collaboration with non-scientific institutions, such as the police, that are capable of providing additional biomaterial and background information from the suspect’s family.

In summary, our novel mathematical framework and its computer implementation provide a possible solution to the long-standing problem of how to properly interpret Y-STR haplotype matches in forensic casework.

## Methods

### Implementation

The importance sampling framework described in the Results section was implemented in a customized Python script (version 3.9)^35^. The software requires three input files, namely (i) the pedigree structure, encoded as a directed acyclic graph (in TGF format), (ii) the k known Y-STR haplotypes (in CSV format) and (iii) the marker-specific mutation rates. The pedigree structure is stored in a *NetworkX* (version 3.1, https://networkx.org/) directional graph (*DiGraph*) object whereas haplotypes and mutation rates are stored in custom class objects. After one of the k males with a known haplotype has been marked as the suspect, the pedigree structure is rearranged as described above by converting the *DiGraph* to an undirected graph (*NetworkX* statement *to_undirected*) and back to a directed graph, using a depth-first search (*dfs_tree*) with the suspect as the root node.

Probability P(h^v^) is calculated by multiple rounds of simulations, but only once per pedigree.

- In each simulation, unknown haplotypes are derived from their (then known) paternal predecessor according to the given mutation rates. The order in which individuals with unknown haplotypes are processed is determined by a level-order traversal (*bfs_layers*) of the rearranged pedigree, i.e. sons of the suspect are processed first, followed by the sons of these sons, etc.
- Individual haplotypes are simulated, and haplotype mutation probabilities calculated, assuming independence of mutations across markers. Only gains and losses of one repeat unit per meiosis per marker are considered in the pilot implementation described here. Other mutations, known to be extremely rare in reality^16,23,36^, are assigned a probability of zero.
- Haplotype simulations are carried out using the *choices* method of the *random* module of Python, each time selecting a mutation type from the set of possible values of -1, 0, and + 1, which represent the loss, no change, or the gain of a repeat unit, respectively. Values are selected with relative weight µ_i_/2, 1-µ_i_, or µ_i_/2, respectively, where µ_i_ is the mutation rate of the i^th^ marker.
- The haplotype mutation probability is obtained as the product of the marker-specific mutation probabilities (i.e. µ_i_/2 or 1-µ_i_) taken over all markers. Once all unknown haplotypes have been simulated, the product of the haplotype mutation probabilities is taken over all edges that were not involved in the simulation of a haplotype.
- Finally, the sought-after probability P(h^v^) is obtained as the average of these products, taken over all simulations.

For a fixed integer 0 < x ≤ n-k, the conditional probability p_x_=P(m(H^u^) = x|h^v^) that exactly x unknown haplotypes match the suspect haplotype is also calculated by way of simulation.

- At the beginning of each simulation of H^u^, x individuals are selected at random from the pedigree members with unknown haplotypes, using the *sample* method of the *random* module of Python. Their haplotypes are set equal to the suspect haplotype. Note that the probability of selecting a given subset of males this way equals the inverse of the binomial coefficient bin(x, n-k), which was calculated using the *combinations* method of the *itertools* module of Python.
- All remaining unknown haplotypes are simulated as described above for the calculation of P(h^v^), following the same level-order traversal.
- The number of simulated haplotypes that match the suspect haplotype is determined at the end of each simulation, and the corresponding proposal weights are summed over those simulations in which this number equals x. This sum of the proposal weights, divided by the total number of simulations, finally provides an estimate of p_x_ for a given value of x.
- The above process is repeated for x running from 1 to n-k. Conditional probability p_0_ is obtained by successively subtracting the p_x_ estimates from unity.

### Software test

Both the importance sampling framework and its Python implementation were tested for a set of generic elementary pedigrees comprising a certain (small) number of typed and untyped males (Fig. 2). In each test, only a single archetypical Y-STR with integer-valued alleles and only one of three different mutation rates µ (0.1, 0.02, or 0.005 per meiosis) were considered. The suspect allele was always set equal to zero. If an elementary pedigree contained typed males other than the suspect, all possible allele assignments to these males were tested, modulo the signs of alleles (Table 1). For example, in pedigree E (Fig. 2), the typed great-grand son of the suspect (male no. 2) could sensibly be assigned only allele + 1 or + 2, but not + 3 because p_1_ and p_2_ would both be zero in this case.

Testing was repeated 10 times for each of the 102 test scenarios (3 mutation rates × 34 allele assignments), with 100,000 simulations performed per repeat, and the resulting estimates of P(h^v^) and p_x_, respectively, were averaged over the 10 repeats. In each repeat, we also determined the percentage difference between the analytical and the simulation-based results. For test scenarios for which the average or the standard deviation of the percentage difference, taken over the 10 repeats per test scenario, exceeded 10%, the whole test of that scenario was re-run with 1 million instead of 100,000 simulations per repeat. Tests were carried out on an Intel^®^ Xeon^®^ Gold 6152 CPU with 22 cores running at a base clock speed of 2.10 GHz, and with 30,976 KB cache per core. For better comprehensibility of the simulation results, and to facilitate performance testing by recording runtime information for the estimation of P(h^v^), only one of the 44 available threads was utilized.

## Data Availability

The present study is based entirely on theoretical considerations and simulated data, the generation of which is described in detail in this published article. Additional information on the simulation process is available from the corresponding author upon reasonable request.

## Software availability

All software developed and used in the present study is publicly available at GitHub (https://github.com/dionzand/match-Y.git).
